# Supplementary material for: The Variance of Photophysical Properties of Tetraphenylethene and Its Derivatives during Their Transitions from Dissolved States to Solid States
Source: Polymers (Basel). 2022 Jul 15;14(14):2880. doi: 10.3390/polym14142880 (PMC9320569; doi:10.3390/polym14142880)
Supplement: Supplementary file 1 [file polymers-14-02880-s001.zip › polymers-1735134-supplementary.pdf]

polymers

Supplementary Materials

In the following discussion, L-TPE denotes the TPE (fully dried powder) dissolved in THF solvent while L-TPE-y (y=1,2, and 3) represents the corresponding TPE-y (fully dried powder) dissolved in ethanol solvent. P-TPE-y (not fully dried powder) signifies the precipitated solid states to directly evaporate L-TPE-y in ambient without any further heat-treatment utilized to accentuate the discrepancy compared to that of TPE-y. The S-TPE-1 (x mg/mL, x=10^{-3}, 10^{-2}, 10^{-1}, 5, and 10) sols were prepared based on the doping ratio x mg of TPE-1:1 mL of TEOS:1 mL of EtOH, and then stirring (200 rpm, IKA® RCT basic) under ambient environment for 24 hours to remove most of EtOH solvent for the following characterizations.

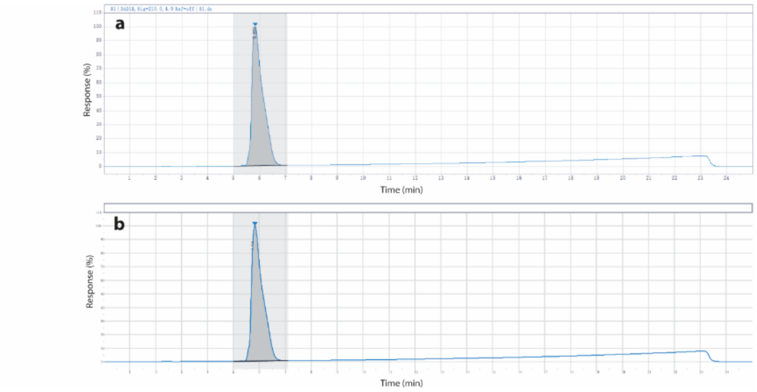

Figure S1. High-performance liquid chromatography results of (a) TPE and (b) benzo[g,p]chrysene measured by utilizing acetonitrile/water (70/30, volume relationship) mixed solvent.

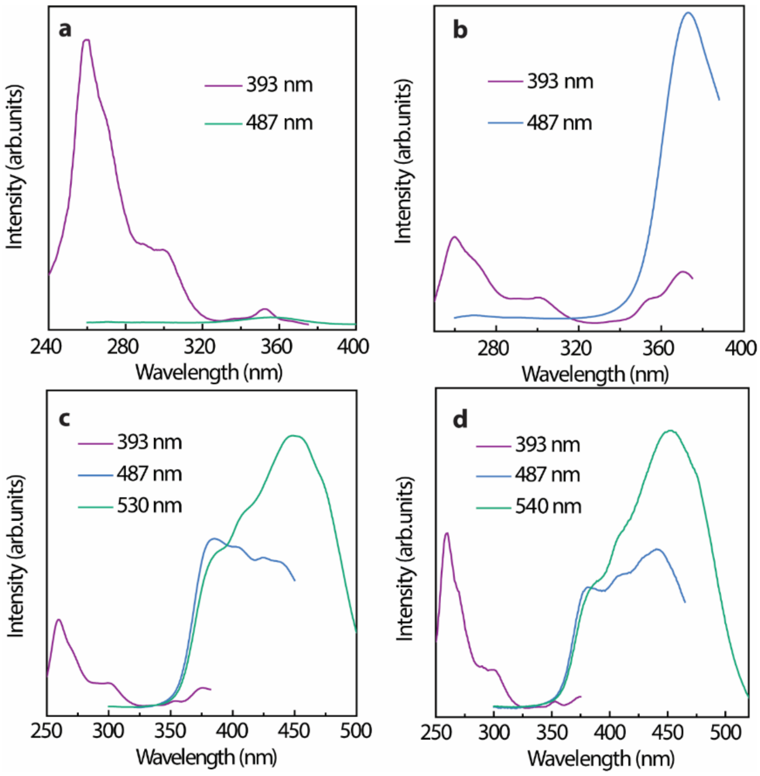

Figure S2. Excitation spectra of L-TPE with concentration (a) 1 mg/mL, (b) 2 mg/mL, (c) 5 mg/mL, and (d) 10 mg/mL in THF solvent, respectively.

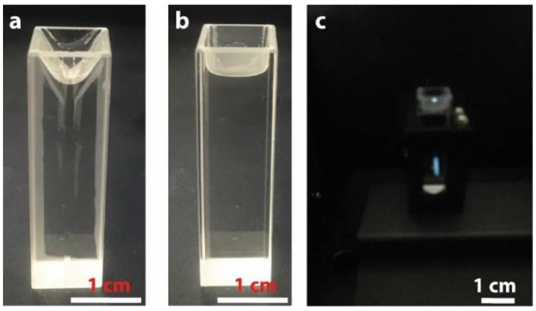

Figure S3. (a) and (b) Photos of TPE in THF solvent with a concentration of 10 mg/mL contained in a quartz cuvette (1 mm light path length) under daylight, and (c) the corresponding photo taken under 280 nm irradiance with the same measurement setting for the corresponding emission spectra.

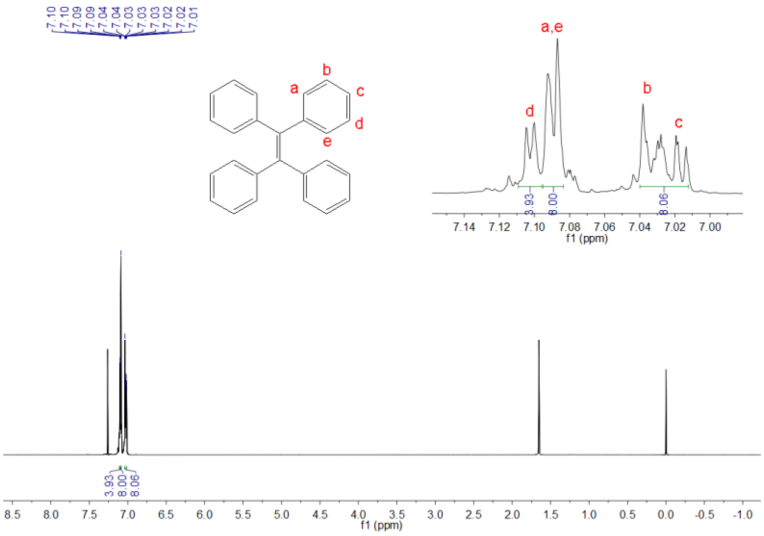

Figure S4. ^{1}H NMR spectrum of TPE in $CDCl_3$ solvent (5 mg/mL).

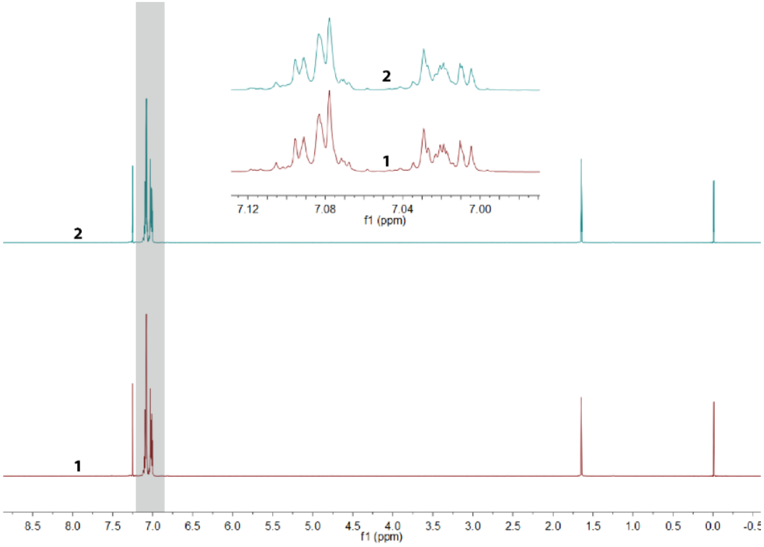

Figure S5. The comparison of TPE ^{1}H NMR spectra in $CDCl_3$ solvent (5 mg/mL) before (curve 1) and after (curve 2) irradiance treatment with the same UV irradiance and exposure time during that of the corresponding excitation wavelength-dependent emission characterization of L-TPE (5 mg/mL).

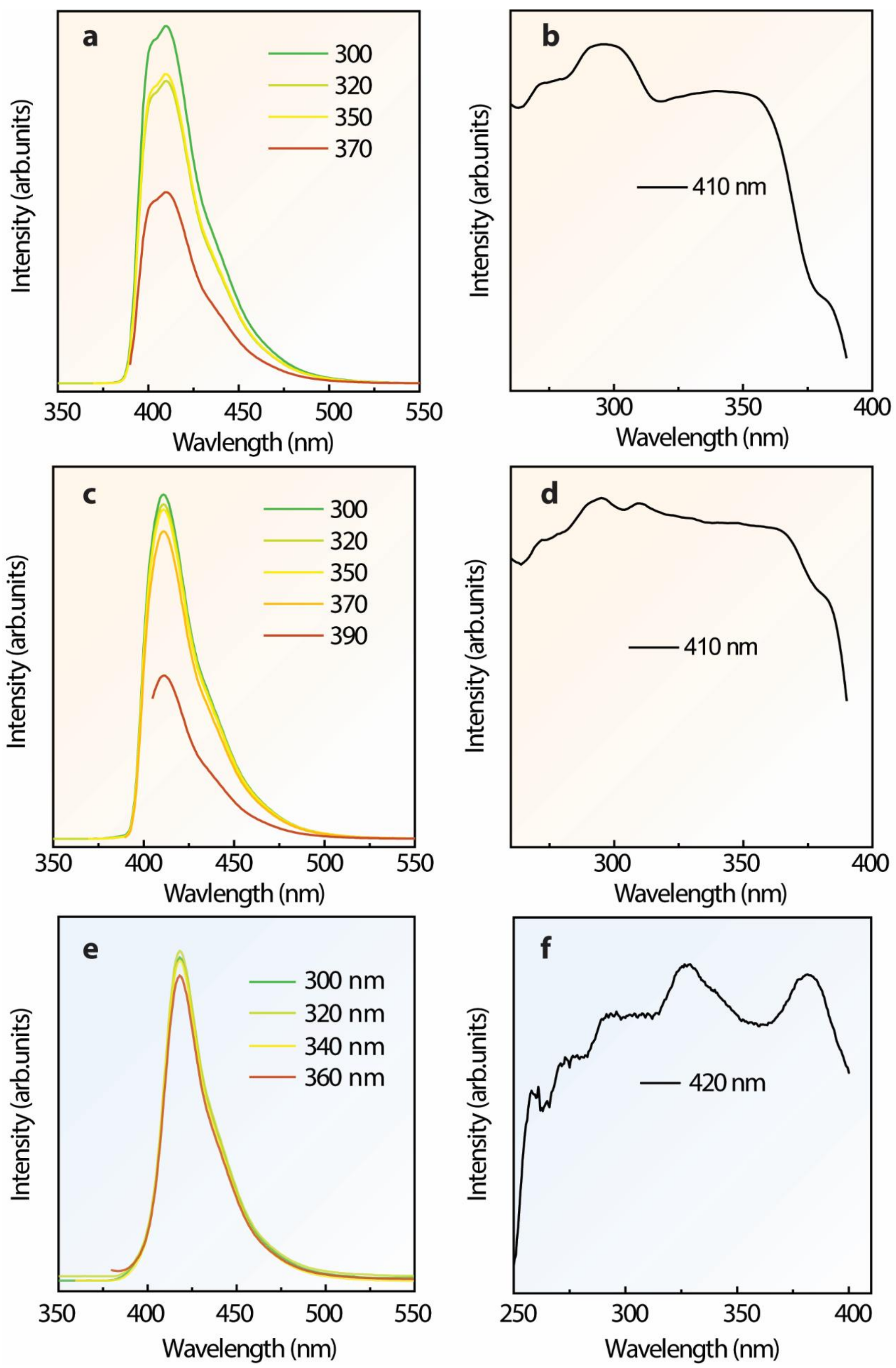

Figure S6. Emission and excitation spectra of benzo[g,p]chrysene in THF solvent with concentration (a)/(b) 1 mg/mL and (c)/(d) 5 mg/mL, respectively (all the measurement operated in the quartz cuvette with 1 mm light path length); and (e) emission and (f) excitation spectra of commercial benzo[g,p]chrysene powder without further treatment.

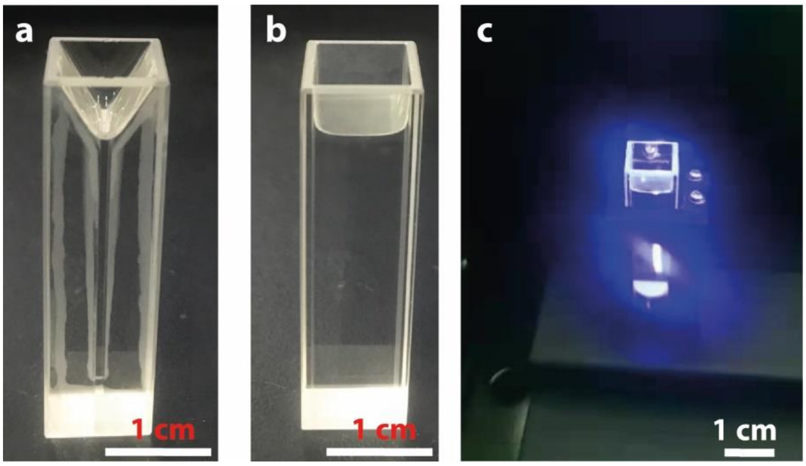

Figure S7. (a) and (b) Photos of benzo[g,p]chrysene in THF solvent with concentration 5 mg/mL contained in a quartz cuvette (1 mm light path length) under daylight, and the corresponding photo (c) taken under 300 nm irradiance with the same measurement setting for the emission spectra.

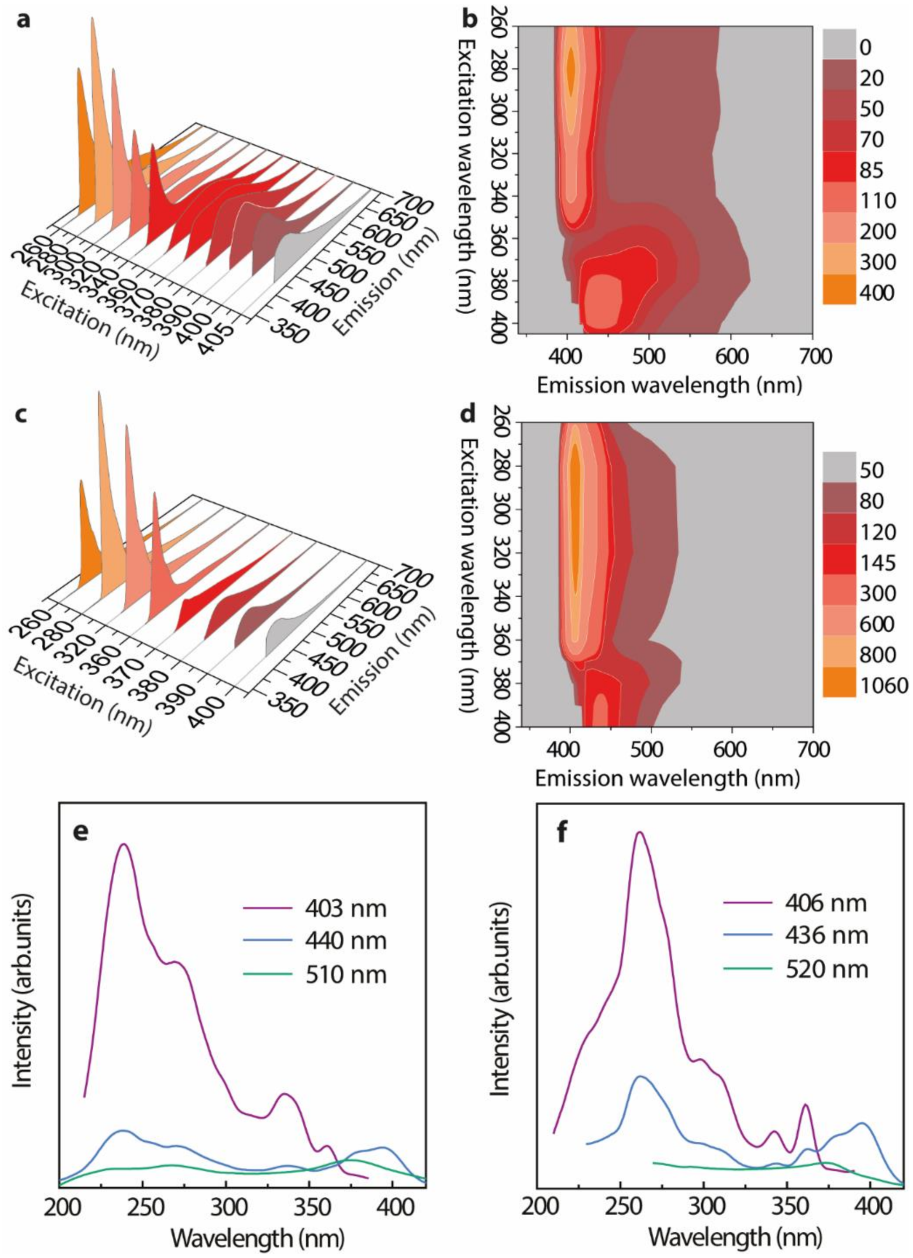

Figure S8. Excitation-dependent three-dimension and two-dimension emission spectra of (a)/(b) L-TPE-1 (5 mg/mL) and (c)/(d) S-TPE-1 (5 mg/mL), and the corresponding excitation spectra of (e) L-TPE-1 (5 mg/mL) and (f) S-TPE-1 (5 mg/mL), respectively.

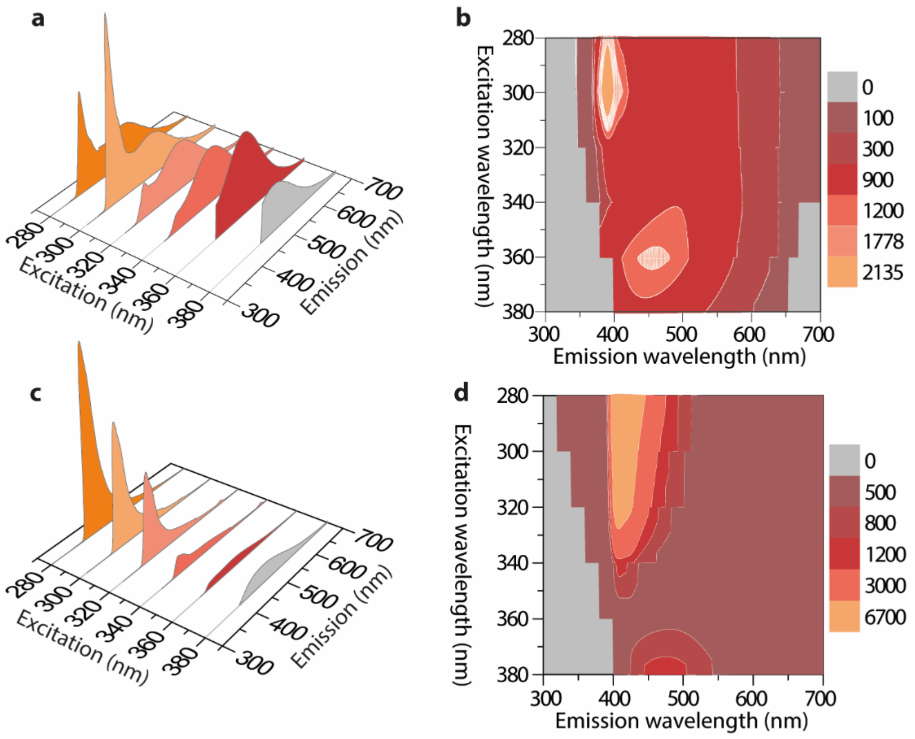

Figure S9. Excitation-dependent three-dimension and two-dimension emission spectra of (a)/(b) L-TPE-2 (2 mg/mL) and (c)/(d) L-TPE-4 (5 mg/mL).

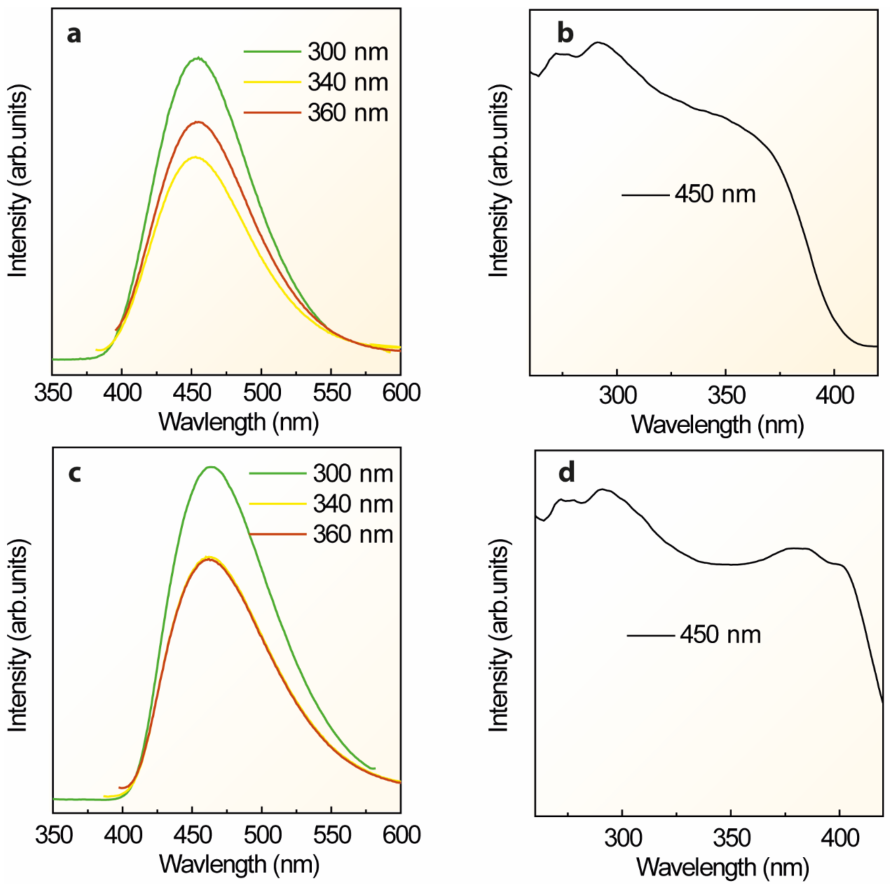

Figure S10. Emission and excitation spectra of (a)/(b) TPE-2 and (c)/(d) TPE-3, respectively.

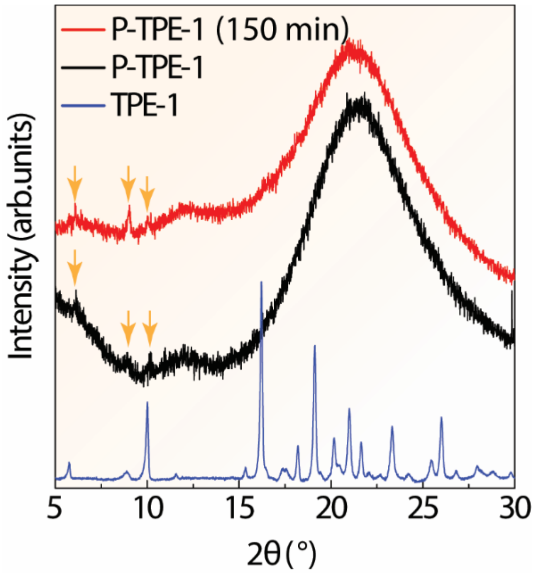

Figure S11. XRD patterns of TPE-1, P-TPE-1 and P-TPE-1 (150 min) processed in an oven under 70 ℃ for 150 min.

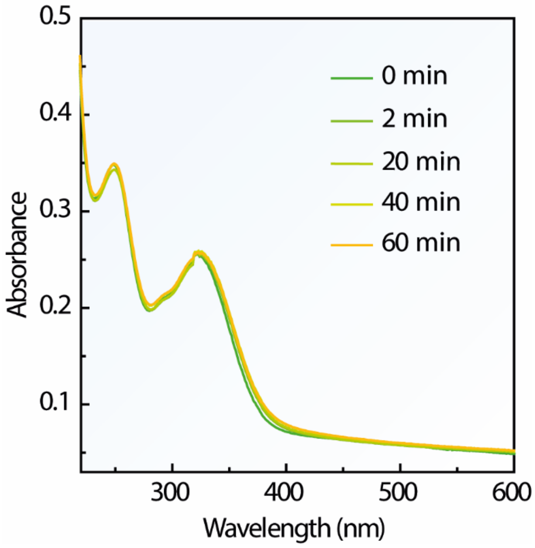

Figure S12. UV–Vis absorption spectra of S-TPE-1 (10 mg/mL) based on heat-treatment time.

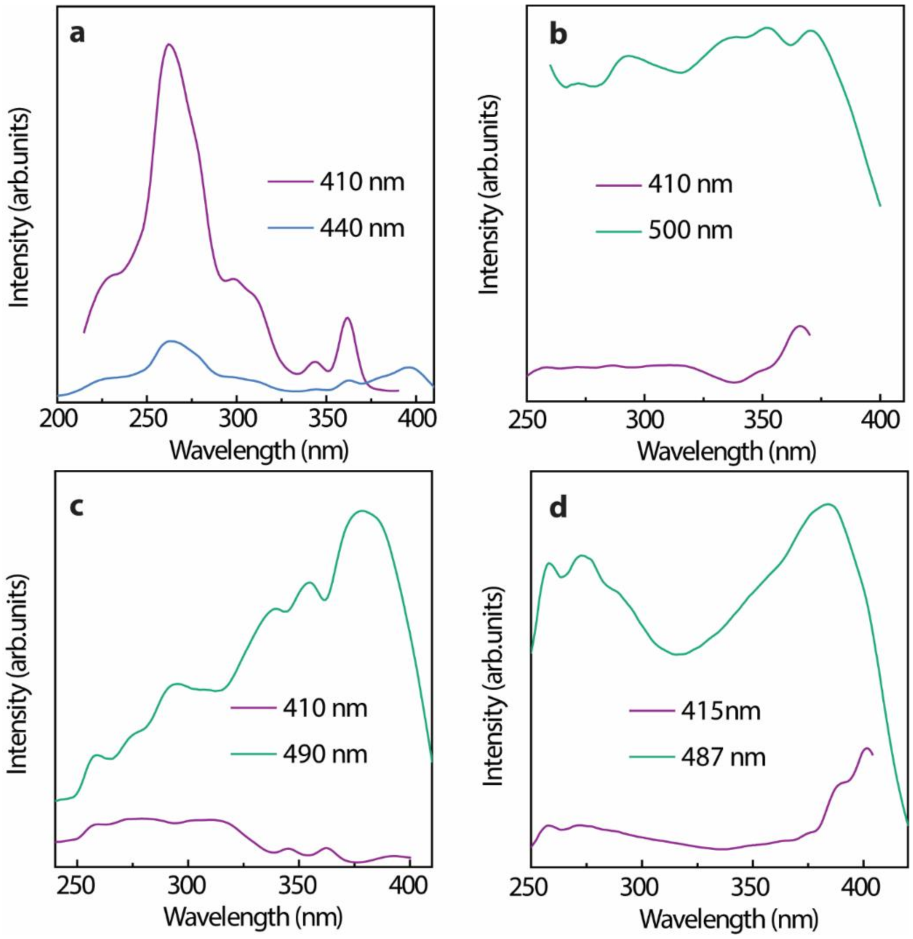

Figure S13. Excitation spectra of S-TPE-1 (10 mg/mL) processed with the 70 ℃ of heat-treatment for (a) 0, (b) 30, (c) 60, and (d) 120 min.

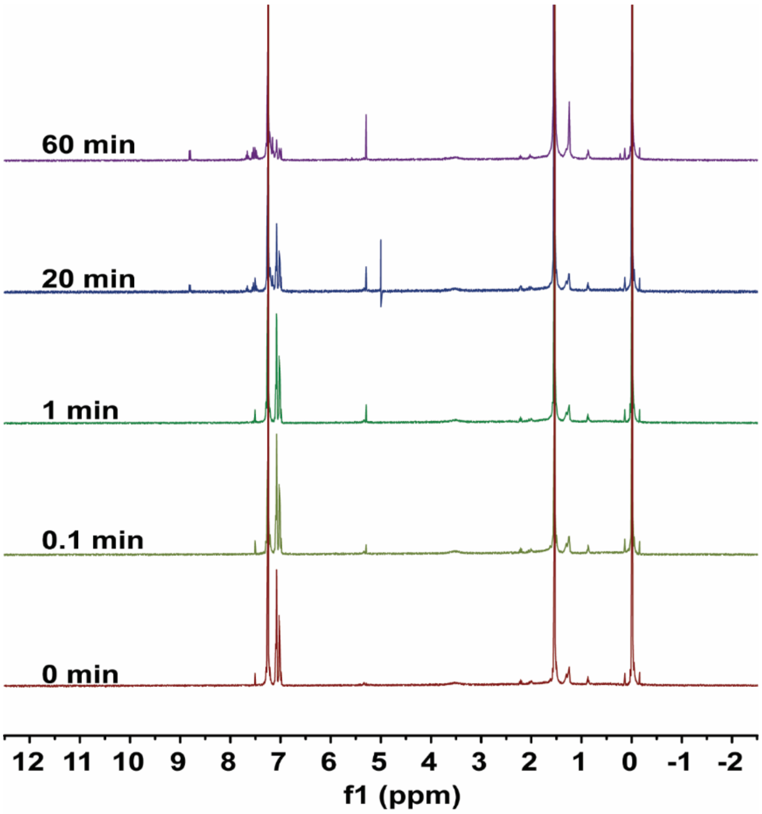

Figure S14. Time-dependent ^{1}H NMR spectra of TPE in $CDCl_3$ solvent (0.1 mg/mL) under continuous intense 280 nm UV irradiance.

Table S1. Corresponding parameters of time-resolution decay curves of Figure 6a and 6b.

λ_{ex}= 300 nm and λ_{em}= 480 nm								
**T (K)**	80	100	150	200	250	260	280	300
**τ (ns)**	5.433± 0.026	5.448± 0.026	5.578± 0.026	5.315± 0.026	3.711± 0.026	3.302± 0.026	2.522± 0.026	1.886± 0.026
λ_{ex}= 300 nm and λ_{em}= 386 nm								
**T (K)**	250	260	270	280	290	300		
**τ (ns)**	0.557± 0.026	0.552± 0.026	0.546± 0.026	0.550± 0.026	0.548± 0.026	0.545± 0.026		
